# Urinary continence recovery after Retzius-sparing robot-assisted radical prostatectomy in relation to surgeon experience

**DOI:** 10.1007/s11701-023-01687-8

**Published:** 2023-08-01

**Authors:** Jorge Fonseca, Gonçalo Froes, Maria Francisca Moraes-Fontes, Jorge Rebola, Rui Lúcio, Miguel Almeida, Ciprian Muresan, Artur Palmas, Ana Gaivão, Celso Matos, Tiago Santos, Daniela Dias, Inês Sousa, Francisco Oliveira, Ricardo Ribeiro, Antonio Lopez-Beltran, Avelino Fraga

**Affiliations:** 1 Unidade de Próstata, Centro Clínico Champalimaud, Champalimaud Foundation, Lisbon, Portugal; 2 Faculté de Médecine et Médecine Dentaire, Université Catholique de Louvain, Brussels, Belgium; 3 Unidade de Imuno-Oncologia, Centro Clínico Champalimaud, Champalimaud Foundation, Lisbon, Portugal; 4 Serviço de Imagiologia, Centro Clínico Champalimaud, Champalimaud Foundation, Lisbon, Portugal; 5 Unidade de Investigação Clínica, Centro Clínico Champalimaud, Champalimaud Foundation, Lisbon, Portugal; 6 Serviço de Medicina Nuclear, Centro Clínico Champalimaud, Champalimaud Foundation, Lisbon, Portugal; 7 Unidade de Anatomia Patológica, Centro Clínico Champalimaud, Champalimaud Foundation, Lisbon, Portugal; 8 Instituto de Ciências Biomédicas Abel Salazar, Universidade do Porto, Porto, Portugal; 9 Instituto de Investigação e Inovação em Saúde, Universidade do Porto, Porto, Portugal

**Keywords:** Caseload, International Consultation on Incontinence Questionnaire Short Form, Learning curve, Prostate cancer, Retzius-sparing robot-assisted radical prostatectomy, Urinary continence recovery

## Abstract

Urinary incontinence is one of the main concerns for patients after radical prostatectomy. Differences in surgical experience among surgeons could partly explain the wide range of frequencies observed. Our aim was to evaluate the association between the surgeons` experience and center caseload with relation to urinary continence recovery after Retzius-sparing robot-assisted radical prostatectomy (RS-RARP). Prospective observational single-center study. Five surgeons consecutively operated 405 patients between July 2017 and February 2022. Continence recovery was evaluated with pad count and by employing the short form of the International Consultation on Incontinence Questionnaire (ICIQ-SF), pre- and postoperatively at 1 year. Non-parametric tests were used. Median age was 63 years, 30% of patients presented with local advanced disease; the positive surgical margin rate (over 3 mm length) was 16%. Complication rate was 1% (Clavien–Dindo > II). One year after surgery, continence was assessed in 282 patients, of whom 87% were pad free and 51% never leaked (ICIQ-SF = 0). With respect to the mean annual number of procedures per surgeon, divided in < 20, 20–39 and ≥ 40, pad-free rates were achieved in 93%, 85%, and 84% and absence of urine leak rates in 47%, 62% and 48% of patients, respectively. Postoperative median ICIQ-SF was five. We acknowledge the limitation of a 12-month follow-up and the fact that we are a medium-volume center. There is no statistically significant association between continence recovery, surgeon’s experience and center caseload. Continence recovery at 1 year after surgery is adequate and robust to surgeon’s experience.

## Introduction

Radical prostatectomy (RP) has been the gold standard surgical treatment in patients with clinically localized prostate cancer and a life expectancy exceeding 10 years [1]. However, with this procedure, urinary incontinence (UI) is one of the main concerns with a harmful effect on quality of life [2]. Robot-assisted radical prostatectomy (RARP) has improved urinary continence (UC) outcomes. A recent systematic review concluded that a surgeon caseload over 50 RP yearly results in improved continence recovery [3]. However, with RARP, UC continues to improve with experience, even after more than 200 cases per surgeon [4].

The recently described Retzius-sparing robot assisted radical prostatectomy (RS-RARP) achieves superior UC outcomes when compared to standard RARP [5]. However, at the present time, it is not clear which factors mostly affect continence recovery in RS-RARP [6]. In general, it is reasonable to assume that less damage to continence-preserving structures, preoperative patient characteristics and surgeon experience may all play an important role.

Of note, different approaches to measure the rate of postoperative UI result in a wide range of frequencies. Pad testing was originally described for measuring female incontinence, involving different methods such as weighing pads and a 1-h pad test. These are, however, cumbersome, inconsistent and dependent on fluid intake, replaced by a simple and convenient “Pad count” method, employed by most studies on post-prostatectomy UC recovery. Nevertheless, one should bear in mind that the “no pad” or “one safety pad per day” definition may exclude a significant proportion of patients who may be incontinent but choose not to wear pads [7]. The International Consultation on Incontinence Questionnaire Short Form (ICIQ-SF) [8] has been consistently used for post-prostatectomy UC recovery evaluation [9]. Henceforth, employing both the ICIQ-SF and pad use has been shown to yield a better insight of postoperative UC and a more accurate demonstration of the differences between surgical techniques [7, 10, 11].

We hypothesized that RS-RARP enhances UC recovery, irrespective of the surgeon's experience and center caseload.

## Materials and methods

### Patients and functional status evaluation

RS-RARP was performed on 405 consecutive patients, operated on by five surgeons, between July 2017 and February 2022, in our comprehensive cancer center. Parameters were collected prospectively, including age and body mass index (BMI), American Society of Anesthesiologists (ASA) score [12], preoperative oncological parameters such as prostate-specific antigen (PSA), highest International Society of Urological Pathology (ISUP) grade group (GG) on biopsy [13], and multiparametric Magnetic Resonance Imaging (mpMRI)-based tumor clinical staging using Prostate Imaging-Reporting and Data System Version 2.1 (PI-RADS v2.1) [14].

At 12 months postoperatively, UC was evaluated by asking the third question of the Expanded Prostate Cancer Index Composite-26 questionnaire as regards pad use [15] as well as the ICIQ-SF questionnaire. The first (ICIQ 1) and the second (ICIQ 2) questions of the ICIQ-SF assessed the frequency and the amount of urine leaked. ICIQ 1 is scored from 0 to 5 (0: never; 1: about once a week; 2: 2–3 times a week; 3: about once a day; 4: several times a day; 5: continuously); ICIQ 2 is scored from 0 to 6 (0: no leakage; 2: a small amount; 4: a moderate amount; 6: a large amount). The third question (ICIQ 3) assesses the effect of leaking on QoL and is scored from 1 to 10 (1: not at all; 10: a great deal). The ICIQ-SF score included the sum of the scores of ICIQ 1 + ICIQ 2 + ICIQ 3 and comprised four severity categories: slight (1–5), moderate (6–12), severe (13–18) and very severe (19–21) [16].

All 405 patients/surgeries were included to compute the surgeons’ experience and center caseload. UC recovery was evaluated in 70% of patients (282/405) for pad use and with the ICIQ-SF questionnaire; missing one or both evaluations (*n* = 87), adjuvant or salvage treatments required before continence was reached (*n* = 33), and pre-operative incontinence according to pad use (*n* = 3) accounted for exclusions. In addition, 14% of patients with a preoperative ICIQ-SF > 0 (41/282) were also excluded from the overall post-operative ICIQ-SF assessment. Pre- and postoperative functional outcomes were not pre-selected and the majority of the first 20 patients operated on by each surgeon had a functional assessment. Every patient provided written informed consent for study inclusion, approved by the Institutional Ethics Committee (Approval 07.07.2017).

### Surgeons’ experience and surgical technique

The surgical team comprised five surgeons who switched from open radical prostatectomy to laparoscopic radical prostatectomy (LRP) in 2014. After a period of proctoring, with the availability of the da Vinci Xi surgical system (Intuitive Surgical, Sunnyvale, CA, USA) in our hospital center from 2016, and a prior experience of 172 LRP procedures, the five surgeons performed RS-RARPs, as described by Galfano [17]. Surgeons exchanged roles in this study: the primary surgeon operated at the console assisted by an assistant surgeon at the patient table.

The surgical procedure as regards neurovascular bundle preservation, lymphadenectomy, blood loss, duration of hospital stay and complications according to the Clavien–Dindo classification were recorded as previously described [18]. An indwelling Foley catheter was kept for at least one week and all patients were considered incontinent in the immediate postoperative period.

### Pathology examination

The same senior pathologist reviewed preoperative prostatic biopsies and surgical specimens. The ISUP consensus and the American Joint Committee on Cancer 8th edition schemes were followed for grading and staging [13]. Pathological parameters included prostate volume, tumor ISUP grade group, tumor stage, surgical margin status and length.

### Statistical analysis

Continuous variables were reported as medians and interquartile ranges (IQR), and categorical variables as counts and percentages.

Three different methods were used to evaluate the association of surgeon’s experience and center caseload with UC recovery at 12 months postoperatively. Firstly, the mean annual number of surgeries performed by each surgeon was computed as a ratio of the number of surgeries individually performed divided by the number of years between the first and the last surgery. Secondly, the surgical experience was divided into classes of 40 surgeries, Class I corresponding to the first 40 surgeries per surgeon, irrespective of the time frame in which they were performed, the subsequent classes (II, III, IV and V) following the same principle. Of note, the surgeon with less than 40 operations was only represented in Class I; the remaining four surgeons are represented in the first and second Classes; the third Class includes data from the two surgeons with higher caseloads; Classes IV and V only include the surgeon with the highest caseload. Lastly, the center caseload was documented by recording consecutive surgeries chronologically.

The percentage of UC recovery achieved per mean annual surgeon`s number of surgeries was calculated. UC recovery proportions and confidence intervals were calculated for each class of experience. Next, cumulative proportions of continent patients were plotted along consecutive surgeries performed in the center.

A statistical significance level of 95% was defined. Statistical analyses were performed using IBM^®^ SPSS^®^ Statistics version 27 for Windows and Microsoft Excel.

## Results

Five surgeons performed RS-RARP on 405 patients. Over the study period, the mean annual number of surgeries per surgeon ranged between 12 and 40, and the center annual caseload varied between 18 and 115 procedures (Table 1). Of note, the surgeon with the least surgeries entered our team during the second half of the study period.

**Table 1:** Number of surgeries performed in the surgical center over the study period, discriminated by the five surgeons

Year	Center	Surgeon 1	Surgeon 2	Surgeon 3	Surgeon 4	Surgeon 5
2017	18	11	7	0	0	0
2018	83	44	22	10	7	0
2019	83	38	16	15	14	0
2020	79	36	13	17	8	5
2021	115	47	28	10	15	15
2022	27	11	10	1	3	2
Total of patients	405	187	96	53	47	22
Mean annual number of procedures per surgeon	87	40	22	15	12	12

The characteristics of the population evaluated for UC recovery were representative of the overall population (Tables 2 and 3). Locally advanced disease (pT3a/pT3b) was present in 30% and ISUP GG ≥ 3 in 21% of surgical specimens. As for the remaining characteristics, 53% of the patients had bilateral nerve sparing, 96% bladder neck preservation, 39% of patients had lymph node dissection and the median of intraoperative blood loss was 200 ml with no requirement for intraoperative blood transfusion. No intraoperative complications occurred. Postoperatively two patients required lymphocele drainage and another two, hematoma evacuation.

**Table 2:** Demographics, disease stage, intraoperative and postoperative data in the full surgical cohort and in the patients subjected to urinary continence assessment

Characteristics	Full surgical cohort	UC assessment cohort
Patients evaluated, *n*	405	282
Age, years, median (IQR)	63 (59–67)	63 (59–68)
Body mass index, kg/m^2^, median (IQR)	27 (24–29)	27 (25–29)
ASA score, median (IQR)	2 (2–2)	2 (2–2)
Preoperative PSA, ng/ml, median (IQR)	7 (5–9)	6 (5–8)
Prostate size, cm^3^, median (IQR)	41 (32–55)	42 (31–35)
Highest biopsy ISUP grade group, *n*	405	282
GG 1, *n* (%)	53 (13)	41 (15)
GG 2, *n* (%)	223 (55)	169 (60)
GG 3, *n* (%)	96 (24)	50 (34)
GG 4, *n* (%)	30 (7)	20 (7)
GG 5, *n* (%)	3 (1)	2 (1)
MRI-based T stage, *n*	392	272
T1, *n* (%)	38 (10)	27 (10)
T2, *n* (%)	254 (65)	182 (67)
T3a, *n* (%)	87 (22)	53 (19)
T3b, *n* (%)	13 (3)	10 (4)
Patients evaluated for preoperative UC, *n*		282
Daily pad use:		
No pad use, *n*		282
Pad use, n		0
ICIQ-SF:		
ICIQ-SF = 0, *n* (%)		241 (86)
ICIQ-SF > 0, *n* (%)		41 (14)
ICIQ-SF score when ICIQ-SF > 0, median (IQR), *n *(%)		4 (3–5)
Nerve sparing, *n*	350	240
No nerve sparing, *n* (%)	60 (17)	40 (17)
Unilateral, *n* (%)	117 (33)	74 (31)
Bilateral, *n* (%)	173 (49)	126 (53)
Bladder neck preservation, *n*	327	224
Preserved, *n* (%)	309 (94)	215 (96)
Not preserved, *n* (%)	18 (6)	9 (4)
Lymph node dissection, *n*	405	282
Lymphadenectomy, *n* (%)	183 (45)	110 (39)
No lymphadenectomy, *n* (%)	222 (55)	172 (61)
Lymph nodes removed, median (IQR)	22 (17–27)	23 (17–27)
Intraoperative blood loss, ml, median (IQR)	200 (100–300)	200 (100–300)
Intraoperative transfusion rate, *n*	0	0
Intraoperative complications, *n*	0	0
Postoperative complications (Clavien–Dindo > II), *n* (%)	5 (1)	4 (1)
Length of hospital stay, days, median (IQR)	2 (2–3)	2 (2–3)
Duration of catheterization, days, median (IQR)	9 (8–9)	9 (8–9)

Shown are the total number of patients evaluated for each parent variable, subsequently followed by the corresponding number and percentage of the sub-variable

*UC* Urinary Continence, *PSA* prostate-specific antigen, *ASA score* American Society of Anesthesiologists score, *ISUP* International Society of Urological Pathology, *MRI* magnetic resonance imaging

**Table 3:** Oncological outcomes and urinary continence recovery in the full surgical cohort and in the patients subjected to urinary continence assessment

Characteristics	Full surgical cohort	UC assessment cohort
Patients evaluated for oncological outcomes, *n*	405	282
ISUP on specimen (grade group):		
GG 1, *n* (%)	37 (9)	27 (10)
GG 2, *n* (%)	261 (64)	197 (70)
GG 3, *n* (%)	98 (24)	53 (19)
GG 4, *n* (%)	8 (2)	5 (2)
GG 5, *n* (%)	1 (0)	0
Pathological T stage (pT):		
pT2, *n* (%)	253 (62)	198 (70)
pT3a, *n* (%)	109 (27)	62 (22)
pT3b, *n* (%)	43 (11)	22 (8)
Surgical margin:		
Overall positive, *n* (%)	148 (37)	90 (32)
Positive, with > 3 mm total length extension, *n* (%)	75 (19)	44 (16)
N stage:		
pNx, *n* (%)	222 (54)	172 (61)
pN0, *n* (%)	147 (36)	96 (34)
pN1, *n* (%)	36 (9)	14 (5)
PSA persistence and recurrence		
PSA ≥ 0.2 ng/ml at 12 months, *n* (%)	30 (7)	15 (5)
Adjuvant and salvage treatments, *n* (%)	61 (15)	13 (5)
UC recovery (12 month post op):		
No pad use, *n* (%)		244 (87)
Pad use, *n* (%)		38 (13)
Patients with preoperative no pad use + ICIQ-SF = 0:		
Postoperative ICIQ-SF = 0, *n* (%)		142 (51)
Postoperative ICIQ-SF > 0, *n* (%)		140 (49)
Postoperative ICIQ-SF score when ICIQ-SF > 0, median (IQR)		5 (4–7)
Patients with preoperative no pad use + ICIQ-SF > 0, *n*		41
Postoperative ICIQ-SF = 0, *n* (%)		15 (37)
Postoperative ICIQ-SF > 0, *n* (%)		26 (63)
Postoperative ICIQ-SF score when ICIQ-SF > 0, median (IQR)		6 (4–10)

Shown are the total number of patients evaluated for each parent variable, subsequently followed by the corresponding number and percentage of the sub-variable

*UC* Urinary Continence, *PSA* prostate-specific antigen, *ASA score* American Society of Anesthesiologists score, *ISUP* International Society of Urological Pathology, *MRI* magnetic resonance imaging, *ICIQ-SF* International Consultation on Incontinence Questionnaire-Short Form

For the oncological outcome measure PSM, the rate was 32%, decreasing to 16% when considering more than 3 mm of PSM total extension length. Adjuvant and salvage treatments were required in 5% of patients and biochemical recurrence at 12 months occurred in a further 5%.

**Fig. 1:**
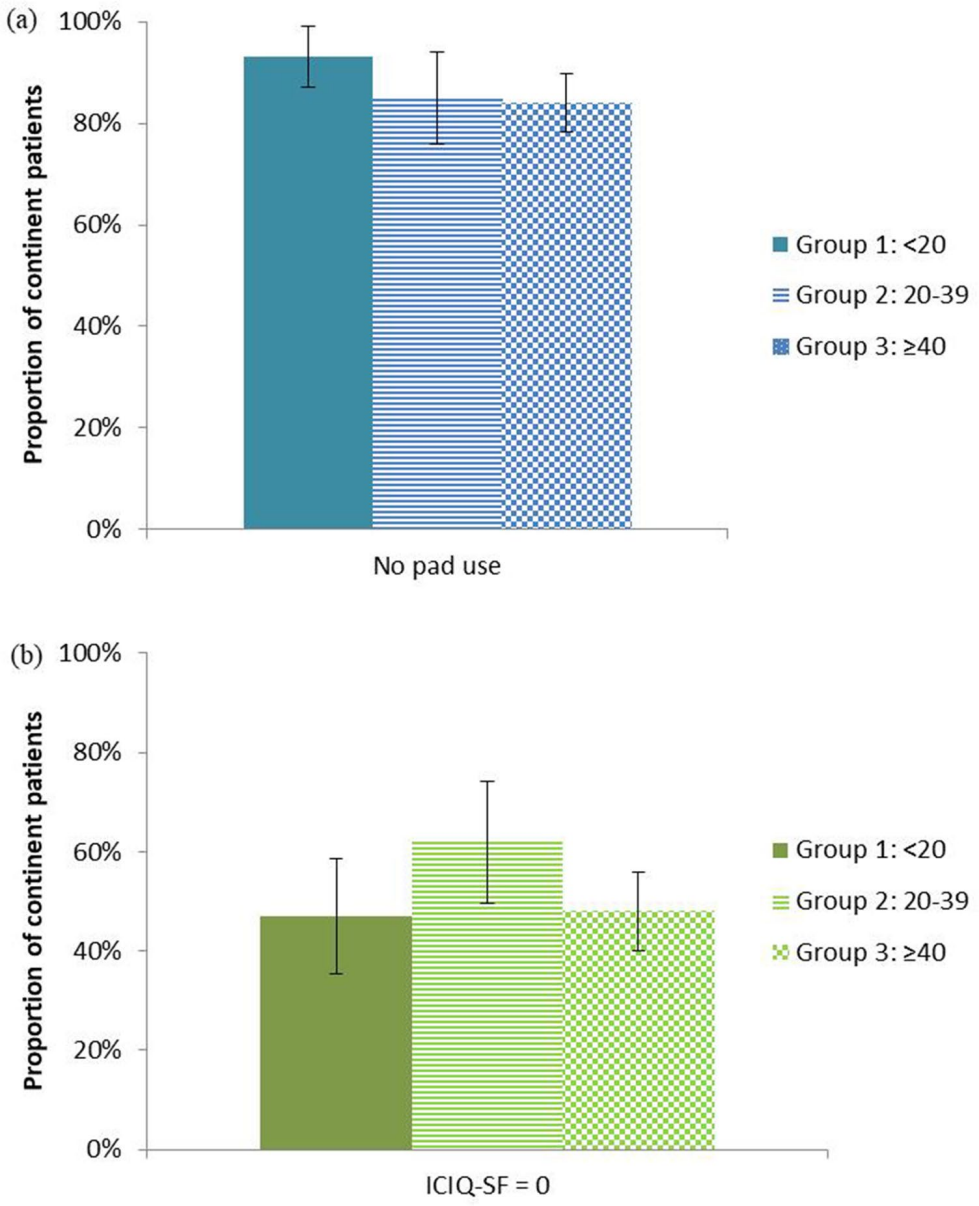
Postoperative UC recovery according to the surgeon’s mean annual number of procedures. Each bar represents the mean annual number of procedures per surgeon divided in three groups (Group 1: < 20; Group 2: 20–39; Group 3: ≥ 40). Group 1 includes three surgeons, whereas Groups 2 and 3 contain one surgeon each. UC recovery is depicted according to pad use (**a**) and ICIQ-SF (**b**). Bars indicate 95% confidence intervals, *UC* Urinary continence, *ICIQ-SF* International Consultation on Incontinence Questionnaire-Short Form

The surgeons’ annual number of surgeries divided in three groups (< 20; 20–39 and ≥ 40) yielded a respective mean rate of UC recovery according to pad use of 93%, 85% and 84% and a mean rate of patients that never leaked (ICIQ-SF = 0) of 47%, 62% and 48% (Fig. 1). No statistically significant differences were observed among the groups, as regards pad use (*p* = 0.226, Chi-square) and ICIQ-SF (*p* = 0.220, Chi-square).

**Fig. 2:**
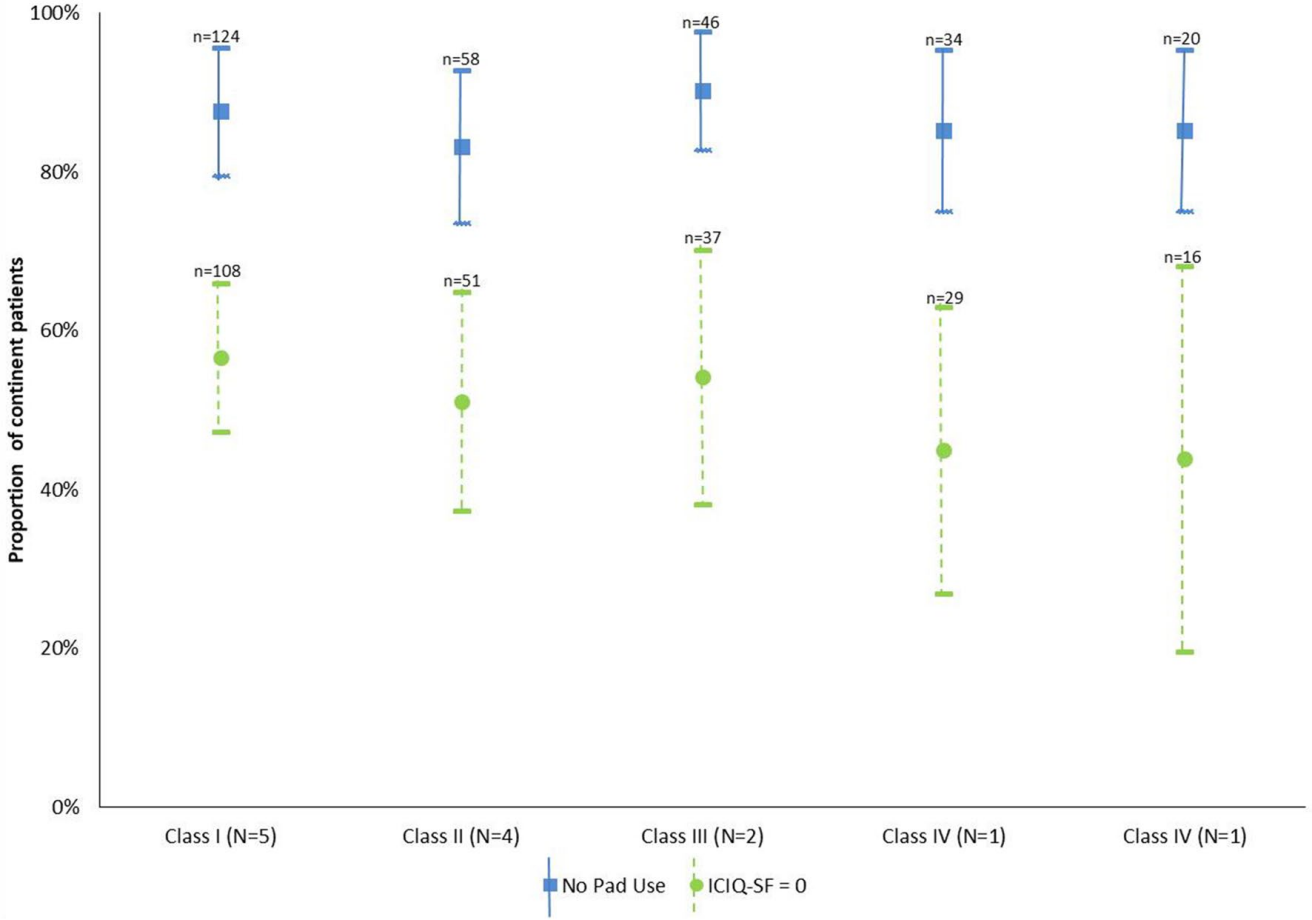
Urinary continence recovery in successive classes of surgeons’ experience according to pad use and ICIQ-SF. Surgical experience distributed into classes of 40 surgeries. Class I corresponds to the first 40 surgeries per surgeon, subsequent surgeries represented in Classes II–V. Squares (pad use) and dots (ICIQ-SF = 0) represent calculated proportions; Bars indicate 95% confidence intervals. *N* represents the number of surgeons in each class; *n* represents the number of patients in each class

According to pad use, UC recovery proportions remain high (> 80%) regardless of classes of surgical experience, as evidenced by overlapping confidence intervals; ICIQ-SF values were lower (roughly 50%) with a slight decline (still above > 40%) in Classes IV and V (Fig. 2). More precisely, at 12 months, according to pad use and ICIQ-SF, the proportion of continent patients was 87% (CI 83–91%) and 51% (CI 43–59%), respectively. Altogether, the range of values with pad use and ICIQ-SF assessments was stable and overlapped irrespective of the surgeon’s experience. In absolute values, post-op median ICIQ-SF for the patients with an ICIQ-SF > 0 was five.

**Fig. 3:**
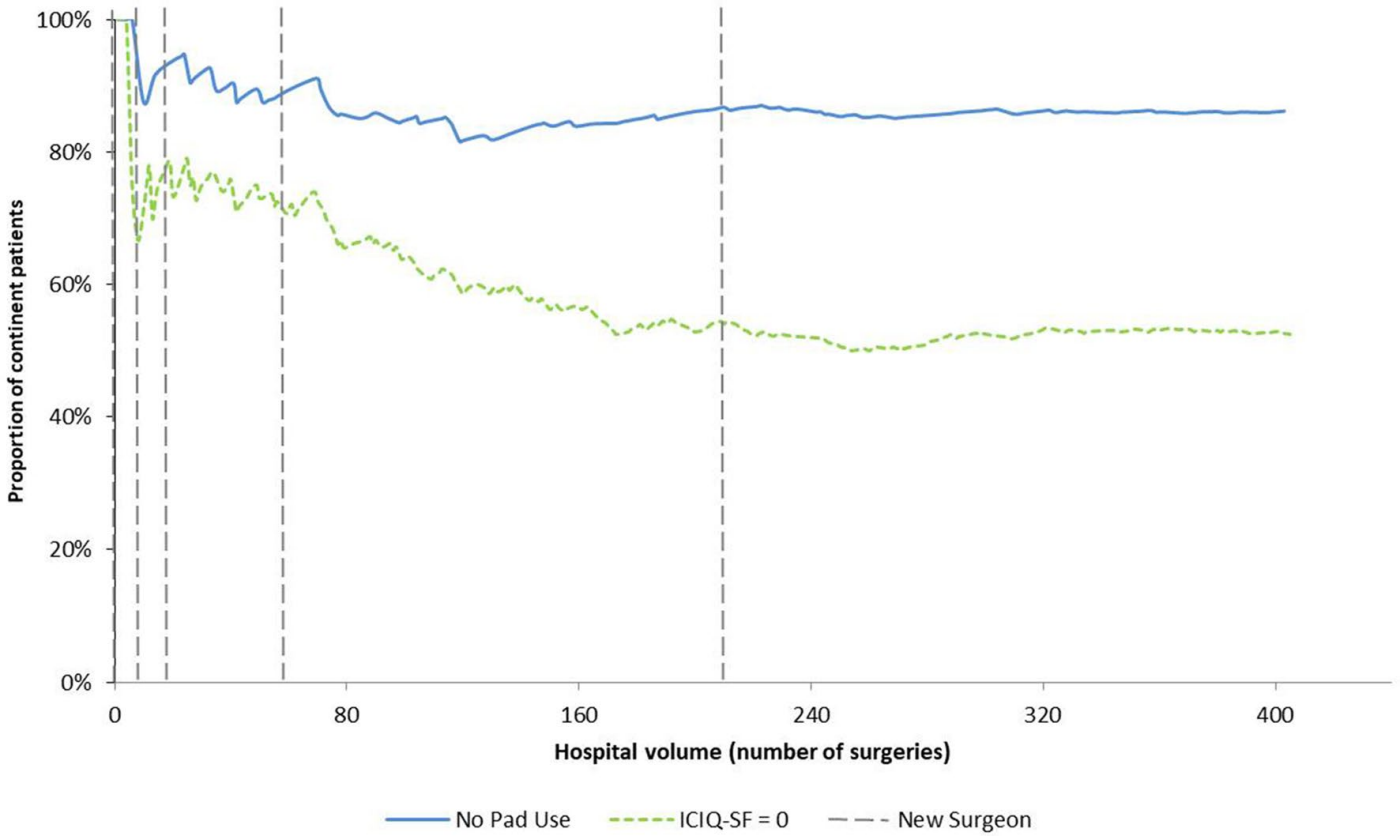
Cumulative urinary continence recovery according to pad use and ICIQ-SF score. Cumulative urinary continence recovery per number of surgeries recorded chronologically. Included are 282 patients enquired for pad use (solid line) and 241 for ICIQ-SF (dotted line). The vertical dotted lines indicate entry points of new surgeons

When considering the center’s increasing caseload, UC recovery was stable according to both pad use and ICIQ-SF, mirroring the trends obtained when analyzing these parameters per surgeons’ class of experience. Furthermore, points of entry of new surgeons did not seem to affect the center’s overall results as regards UC recovery (Fig. 3). We highlight that 15 out of 41 patients (37%) that were continent according to pad use but had a preoperative ICIQ-SF > 0, reported no urine leak at 12 months postoperatively (ICIQ-SF = 0).

## Discussion

Processes of learning related to a surgical technique can be recorded in two ways: measures of surgical process, and measures of health impact. For example, as regards RP, surgical measures include operative time, blood loss, and technical adequacy of cancer resection; health impact measures include, amongst others, morbidity rates. Across health settings, when learning a new procedure, it seems reasonable to expect that performance tends to improve with experience, and graphically plotting performance against experience produces a learning curve. The curve should allow for the establishment of threshold volumes above which favorable patient outcomes can be expected. This has remained somewhat controversial due to the complexity of the underlying condition and the relative roles of individual clinicians versus unit or hospital volumes [19]. Moreover, the advent of robotic facilities and new surgical routes have facilitated otherwise complicated and technically demanding procedures, theoretically allowing for skills to be swiftly acquired and for a plateau phase to be rapidly achieved, removing the expected initial steepness effect of the learning curve. Accordingly, in this study focusing on UC recovery post RS-RARP, whichever way it was measured, there was no initial ascending phase in the learning curve with respect to UC recovery, neither as a function of the surgeons’ experience, nor as regards the center caseload.

Improving UC and other patient outcomes after prostatectomy has been an ongoing challenge with unceasing rises in continence rates accompanying technological and surgical innovation. At 12 months postoperatively, a meta-analysis revealed a wide variation in post-RARP UI using a “no pad” definition, ranging from 4 to 31%, with a mean value of 16% [20]. Subsequent studies reported post-RARP UC recovery rates of 60% [10] and 79% [7]. Still according to pad use, UC recovery for RS-RARP at one year postoperatively has been reported at 96% [21] and 86% [22]. With our strict “no pad-use” UC recovery definition, our pad-free rate of 87% is very near these optimal results.

According to ICIQ-SF, in the worst-case scenario, 70% of patients subjected to RP remained incontinent at one year after surgery [9]. The rates have improved for RARP, reporting UC between 30 and 46.8% [7, 9]. According to the ICIQ-SF score, the severity of incontinence in these studies ranged from slight to moderate [11]. Accruing and centralizing surgical experience have been promoted as a mean to improve patient outcomes [23]. Our literature search failed to reveal the severity of incontinence on patients after RS-RARP, however, a recent case series reported slight incontinence in 50 patients, with a mean ICIQ-SF value of 3.5 [22]. In our series, 51% of our patients never leak when assessed with the ICIQ-SF at 12 months, and in those who leak, the median ICIQ-SF is 5 (ranging from 4 to 7), indicating slight to moderate impairment on the quality of life.

The rate of positive surgical margin (PSM) in our series is 32%. Yet, the multifocal PSM rate, which is predictive of biochemical recurrence and requirement for secondary treatment is much lower, standing at 16%. Our results should be interpreted in the light of a lower clinical estimate of locally advanced disease (23%) leading to a more preserving nerve-sparing approach, when in fact, retrospectively, 30% of patients had pathologically confirmed locally advanced disease. Altogether, our rates compare favorably with other series in terms of oncological outcomes [24, 25].

The main limitation of our study is the low number of overall surgeries. However, it is precisely the fact that there are surgeons with different level of experience that allows for a comparison between classes of experience. There is also a steady rise in the number of surgeries performed, alike other surgical centers where robotic surgery has been introduced [26].

At 12 months after RS-RARP UC recovery was similar between experienced and less experienced surgeons and when the center results were analyzed cumulatively. Once again, our  UC results are in line with other series. UC recovery measured by pad count was excellent. However, when UC was measured by ICIQ-SF, even though ranging from slight to moderate, approximately half of the patients still suffered from incontinence at 12 months postoperatively.

While RS-RARP UC recovery measured through pad use seems robust to surgeons’ experience, we acknowledge that measurement through pad use is a method that is vulnerable to symptom minimization. The decoupling of evaluation methods has an important consequence: it highlights the fact that even though RS-RARP results are much improved when compared to RP, full UC recovery remains an unmet need.

## Data Availability

Data supporting the results is available upon reasonable request.
